# Linking *vgll3* genotype and aggressive behaviour in juvenile Atlantic salmon (*Salmo salar*)

**DOI:** 10.1111/jfb.15040

**Published:** 2022-03-29

**Authors:** Paul Bai Bangura, Katriina Tiira, Petri T. Niemelä, Jaakko Erkinaro, Petra Liljeström, Anna Toikkanen, Craig R. Primmer

**Affiliations:** ^1^ Organismal and Evolutionary Biology Research Programme, Faculty of Biological and Environmental Sciences University of Helsinki Helsinki Finland; ^2^ Lammi Biological Station University of Helsinki Lammi Finland; ^3^ Natural Resources Institute Finland (Luke) Oulu Finland; ^4^ Institute of Biotechnology Helsinki Institute of Life Science (HiLIFE) Helsinki Finland

**Keywords:** aggressiveness, Atlantic salmon, energy, maturation, *vgll3* genotype

## Abstract

We tested the possibility that *vgll3*, a gene linked with maturation age in Atlantic salmon (*Salmo salar*), may be associated with behaviour by measuring aggressiveness and feeding activity in 380 juveniles with different *vgll3* genotypes. Contrary to our prediction, individuals with the genotype associated with later maturation (vgll3*LL) were significantly more aggressive than individuals with the genotype associated with earlier maturation (vgll3*EE). Individuals with higher aggression were also significantly lighter in colour and had higher feeding activity. Although higher aggression was associated with higher feeding activity, there was no association between feeding activity and *vgll3* genotype. Increased aggression of vgll3*LL individuals was independent of their sex and size, and genotypes did not differ in their condition factor. These results imply that aggressive behaviour may have an energetic cost impairing growth and condition, especially when food cannot be monopolized. This may have implications for individual fitness and aquaculture practices.

## 1 INTRODUCTION

Resource acquisition is an important component of individual fitness. In most habitats, food supply is limited to some extent. In these instances, individuals must compete for access to the resources necessary for survival. Competition over access to scarce resources can drive organisms to manifest aggressive behaviour (Colleter & Brown, 2011). Consistent with the notion that many types of behaviours have genetic components (Boake, 1994; Dochtermann *et al*., 2019; Foster & Endler, 1999), aggressive behaviour has also been suggested to be at least partially influenced by genetics in a wide range of taxa, including insects (Dierick & Greenspan, 2006), mammals (Kukekova *et al*., 2011; Miczek *et al*., 2001) and fishes (Laine *et al*., 2012; Tiira *et al*., 2003). Aggression is considered as a good predictor of social dominance – an individual's tendency to win contests and dominate resources (Nakano, 1994; Wilson *et al*., 2011). Thus, social hierarchies that can form as a result of aggressive behaviour can result in inequalities for access to food amongst individuals within a population and contribute to variation in individual growth rates (Jobling & Wandsvik, 1983; Martins *et al*., 2008; Montero *et al*., 2009; Vera Cruz *et al*., 2006) and thereby fitness (Huntingford & Turner, 1987).

In general, aggressiveness is considered as being advantageous for individuals, as it promotes social dominance and thus greater access to food, potentially resulting in increased performance and survival of the individual (Tiira *et al*., 2003). However, individuals exhibiting higher aggression levels also have higher energy demands, as aggressiveness results in both greater energy loss due to movement involved in conflicts as well as injuries resulting from such conflicts (Ang & Manica, 2010). This means that it is likely beneficial for individuals to display aggression only when necessary, as opposed to maintaining aggressiveness constantly. This has been demonstrated, for example, in salmonid fish juveniles, with aggression observed as a situational behaviour rather than a constant one (Li & Brocksen, 1977). Furthermore, excessive aggression may incur metabolic costs (Puckett & Dill, 1985). Thus, although aggressiveness has benefits (Biro & Stamps, 2008; Stamps, 2007; Wilson, 2013), these must be weighed against the potentially negative effects that high aggression levels can cause.

Atlantic salmon juveniles display aggressiveness and form dominance hierarchies also in captivity. A number of studies have shown a positive correlation between aggressiveness and growth in salmonid juveniles (Metcalfe *et al*., 1990; Nicieza & Metcalfe, 1999), suggesting that aggression may have net‐positive benefits in this teleost family. In Atlantic salmon, growth rate is known to be linked with the age at which individuals reach sexual maturity, with initially faster growing juveniles being more likely to mature earlier (Debes *et al*., 2021; Jonsson & Jonsson, 2007). Age at maturity has also been shown to have a genetic basis, with genotypes at the transcription co‐factor vestigial‐like family member 3 (*vgll3*) locus having a large effect on the age, and thereby also the size, at which Atlantic salmon mature, accounting for up to 39% of observed variation in the trait (Ayllon *et al*., 2015; Barson *et al*., 2015). Recently, Debes *et al*. (2021) observed that the influence of the *vgll3* locus on maturation time appears to be mediated by body condition, namely, the relative mass of an individual given its length, reflecting individual fatness (Sutton *et al*., 2000). More specifically, Debes *et al*. (2021) observed that juvenile Atlantic salmon harbouring genotypes linked with earlier maturation (vgll3*EE) had a higher condition factor than their vgll3*LL counterparts (the genotype linked with later maturation) in a common garden environment, but there was no association between *vgll3* genotype and length. Males with the vgll3*EE genotype also matured more frequently, but also their nonmaturing vgll3*EE female siblings had a higher body condition factor, suggesting that the higher condition was due to alternative processes rather than a result of maturation *per se*. However, the underlying mechanisms linking *vgll3* with body condition and maturation remain unclear. Given that (*vgll3*‐dependent) variation in body condition and maturation may be driven by variation in resource input, we hypothesized that these links might be mediated by genotype‐dependent behavioural aggressiveness so that vgll3*EE individuals express the highest aggressiveness, thus providing greater access to food. The aim of this study was therefore to investigate the effect of *vgll3* genotype on aggressive behaviour in juvenile Atlantic salmon using controlled aquarium‐based trials.

## 2 MATERIALS AND METHODS

### 2.1 Fish husbandry and maintenance

Individuals used in this study were derived from a first‐generation hatchery‐maintained Neva River strain at the Natural Resources Institute Finland (62°24′50′′N, 025°57′15′′E, Laukaa, Finland). Eggs and milt of individuals with known *vgll3* genotypes were transferred to facilities at the University of Helsinki in October 2019. Families were created by crossing vgll3*EE males with vgll3*EE females and vgll3*LL females with vgll3*LL males so that all offspring within a family had the same homozygous *vgll3* genotype. Fertilized eggs were incubated in replicated family compartments in vertical incubators with a re‐circulating water system with a constant water temperature of 7°C until March 2020 as in Debes *et al*. (2021). They were then transferred to facilities at the Lammi Biological Station (61°04′45′′N, 025°00′40′′E, Lammi, Finland) as alevins several weeks before they commenced feeding independently. In total, seven families of salmon fry where all offspring had the genotype vgll3*EE and seven families where all offspring had the genotype vgll3*LL were used in the study. Each family (60–200 individuals per family) was reared in a separate circular tank (50 cm height × 90 cm diameter × water depth 21 cm) to maintain family identity. Tanks received continuous flowing filtered water from a nearby lake with an average dissolved oxygen level of 11.2 mg l^−1^, pH 7.13, conductivity 88.1, N/NO_2_ + NO_3_ 1151 μg l^−1^, N/NH_4_ 4 μg l^−1^ and the lake's natural water temperature was consistently increased by 1°C using an external heater to promote faster growth. The average daily water temperature for the experimental period was 5.58°C (range 4.5–7.7°C). Individuals experienced ambient light conditions (fluorescent lights evenly spaced along the tanks at a height of 50 cm above the top of the tank) similar to the local light cycle [ranging from 10 h light (L):14 h dark (D) at the beginning to 18 h L:6 h D at the completion of the experiment]. Each tank contained 12 rocks, 10–15 cm in diameter, that served as refuge for the fish and thus provided a possible stimulus to fight for advantageous locations. Fish were initially fed with commercial 0.2 mm pellet food (Vita, Veronesi, Verona, Italy) eight times daily using automated feeders. As fish grew, a portion of 0.5 mm pellets was added and this proportion was increased over time to meet the growth transition of fish. Tanks were flushed of uneaten food daily, were cleaned regularly, and dead individuals were removed and recorded daily. The mortality rate throughout the study was 3.9% and the average mass of individuals used in trials was 0.42 g and average length was 3.6 cm.

### 2.2 Behavioural trial setup

Twenty‐seven identical experimental glass aquaria (30 cm wide × 25 cm deep × 40 cm high) filled to a depth of 30 cm with water flow of approximately 3 l min^−1^ were used for the aggression trials. The water in‐flow point in each tank was positioned in a such a way to create a single beneficial territory area near the inflow. A floating feed ring (5 cm) was placed on the surface middle section of each aquarium so that food pellets would drift along with the water current, thereby further enforcing a profitable territory location. The aquaria were covered on three sides to minimize disturbance, and the top was covered with polystyrene to prevent the fish from jumping from the aquaria. Enrichments were not provided in the experimental aquaria to ensure proper observation of fish, but fish were able to escape aggressions due to the size of the aquarium. Photoperiod and water source were the same as those of the holding tanks described above.

### 2.3 Behavioural trials

To evaluate aggressiveness, behavioural trials were conducted in the above‐described aquaria using four approximately size‐matched individuals (mean within‐trial length difference 0.31 ± 0.17 cm), two of each *vgll3* homozygote genotype, selected from four randomly chosen families of the 14 available (the four individuals within a trial were always from different families). Prior to the trials, individuals were netted from the communal holding tanks and anaesthetized (sodium bicarbonate‐buffered methanesulfonate 125 mg l^−1^) in a separate bath until they did not respond to touch for measurements and tagging. For visual identification during the behavioural observations, each fish was tagged with a randomly chosen yellow, red, orange or blue mark in the dorsal musculature [visible implant elastomer (VIE) tags, Northwest Marine Technology, Anacortes, USA], and length and mass recorded. Following recovery from the anaesthetic (approximately 15 min), the four size‐matched fish to be included in the specific trial were placed in one of the 27 test aquaria and allowed to acclimate for at least 12 h without food before behavioural observations commenced (Lahti *et al*., 2001; Tiira *et al*., 2003).

All 27 aquaria were used simultaneously in the experiment during each session. Individuals within an aquarium were unfamiliar with the aquarium prior to the acclimation period, and individuals were also unfamiliar with each other as they had been kept in different (family‐specific) tanks prior to the experiment. Interobserver calibration for recording of behaviours was conducted at the beginning of the experiment following initial training, whereby each observer recorded behaviours of the same set of four fish for 30 min each and then behaviour recordings were compared. Fish were observed from behind cloth blinds by an observer not familiar with the *vgll3* genotypes of the individuals. Each aquarium was observed for a total period of 30 min per day over three consecutive days by one of three observers, with the observer of a specific aquarium changing each day. As food is known to stimulate aggressive behaviour (Newman, 1956), fish were fed 0.5 g of pellet food (0.5 mm) at the start of the observation period in a feeding ring in the same location, thereby defining a profitable feeding territory to defend in the aquarium. The number of aggressive behaviours received and performed (nips, charges, chases; Keenleyside & Yamamoto, 1962) and approaches (Symons, 1968) as well as the number of food eating attempts (hereafter *feeding activity*) was recorded for each individual. *Actual aggression* was calculated as the total number of aggressive behaviours an individual performed towards any of the other three individuals. *Net aggression* was calculated as actual aggression minus the number of aggressive behaviours an individual received towards it from any of the other three individuals. Furthermore, as colour change has been shown to occur regularly during agonistic behaviour with aggressive fish becoming lighter coloured while submissive fish show more striking dark colour (Keenleyside & Yamamoto, 1962), the colour of individuals was scored at the end of each observation day. Normal brown (neutral colour; Keenleyside & Yamamoto, 1962) individuals were awarded 0 points, light‐coloured individuals were awarded 1 point and dark‐coloured individuals were awarded −1 point. Water temperature was also recorded at the beginning of each day. The Fulton's condition factor for each individual was calculated based on the mass and length of each individual at the beginning of the trials as shown in Equation (1), where *W* is weight (in g) and *L* is length (in cm).

(1) $$\text{Condition factor}={\left(\frac{W}{L}\right)}^{3}\times 100$$

There were no mortalities or visible injuries suffered by individuals during the trials. At the end of each trial (three 30 min observation sessions over three consecutive days), the four individuals in each of the 27 aquaria were removed, euthanized with an overdose of sodium bicarbonate‐buffered methanesulfonate (250 mg l^−1^) and a fin sample was stored in 95% ethanol for *vgll3* genotype confirmation and sexing. Overall, four sessions were conducted in each of the 27 aquaria [in total 108 trials, including a total of 432 individuals (7–40 per family)]. However, post‐trial genotyping of all individuals identified a small number of cases where individuals with an incorrect *vgll3* genotype had been included in a trial, resulting in the exclusion of 13 trials from the data‐set, leaving 95 trials (380 fish) for analyses. Each aquarium trial was observed for a total of 90 min (three 30 min sessions across three consecutive days). The three sessions in each trial were conducted consecutively, and thus the entire experimental period was 12 days.

### 2.4 Statistical analysis

The effect of *vgll3* genotype on aggressiveness was tested using actual and net aggression scores as the dependent variable in separate univariate mixed effects models. We used a frequentist framework for actual aggression and a Bayesian framework for net aggression (since frequentist models did not converge well for net aggression). Bayesian models sampled two Markov chains, each with 2000 iterations, with a sampling interval of two. For both traits, we fitted genotype (categorical fixed effect: vgll3*EE & vgll3*LL) and observer (categorical fixed effect: three levels) as covariates. Sex was tested as a potential covariate but it had negligible effect on model fit (*P* approximately 0.99) and was excluded from the final models. Random effects included family (*n* = 14), aquarium (*n* = 27), individual identity (*n* = 380) and day of observation (*n* = 12), with four observation sessions (*n* = 4) and trial number (*n* = 95). We additionally tested whether aggression was associated with fish colour (dark, neutral, or light) and feeding activity (continuous) by including them in the above‐described models as covariates. We tested whether genotype was a significant predictor of either feeding activity or colour as response variables, again including all covariates and random effects described above, except for trial number, which overparameterized the model. Finally, condition factor was tested as a continuous response variable in association with predictors of genotype, observer and all the random effects described above.

Statistical modelling was carried out using R version 3.6.1, with packages glmmTMB v1.0.2.1 (actual aggression) and brms v2.15.0 (net aggression). For actual aggression, we used quadratic negative binomial error distribution to account for the large number of zero scores. For net aggression, we used a Gaussian error distribution. Significance was determined by using a *P* value threshold of 0.05 in the case of actual aggression and a 95% credible interval [95% confidence interval (CI)] excluding zero in the case of net aggression (due to Bayesian modelling). Normality of residuals was verified using QQ plots and homogeneity of residuals across predictor levels was confirmed. Multicollinearity was deemed negligible given the low variance inflation factor of each predictor (all below 1.03).

### 2.5 Ethical statement

The care and use of experimental animals complied with the European Union Directive 2010/63/EU animal welfare laws, guidelines and policies as approved by the Animal Experiment Board in Finland (ELLA) under Licence SAVI/42575/2019.

## 3 RESULTS

### 3.1 Aggression versus genotype

**FIGURE 1:**
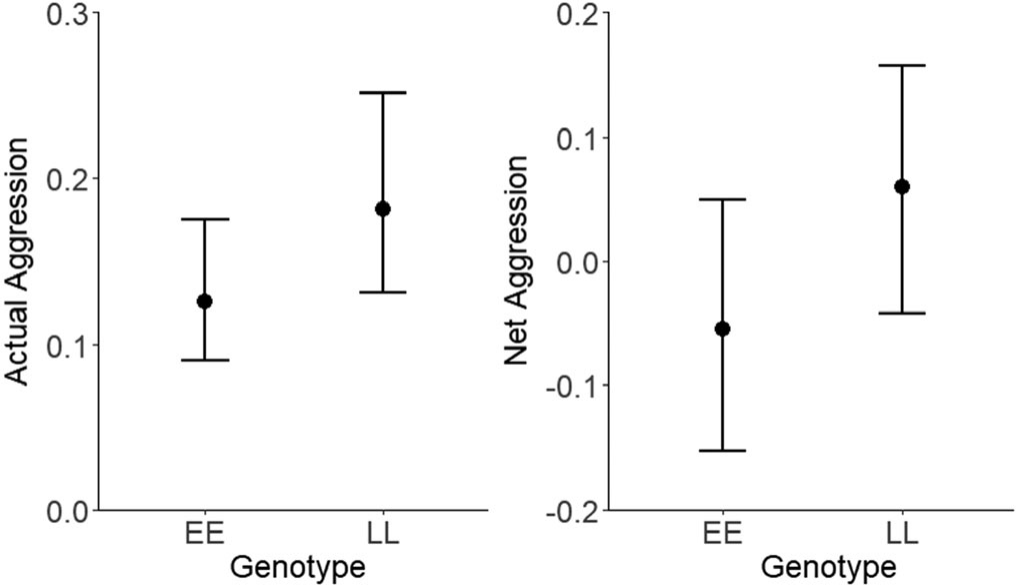
Predicted actual and net aggression scores by *vgll3* genotype from each model at the data scale. Error bars denote 95% confidence intervals (left) or 95% credible intervals (right)

For both actual and net aggression, the vgll3*LL genotype was associated with significantly higher aggression scores than the vgll3*EE genotype (Table 1 and Figure 1). There was an observer effect for actual aggression, but not for net aggression (Table 1). Closer assessment of the records of each observer indicated that observer 1 recorded fewer aggression observations overall compared to either observer 2 or observer 3 (observer 1 average aggressions recorded: 0.12 *vs*. 0.26 for observer 2 and 0.19 for observer 3). We therefore reanalysed the data excluding the measurements of observer 1 to ensure that they were not affecting our conclusions. When excluding data from this observer, and controlling for the same covariates and random effects, the direction of the genotype effect was the same, and significant differences were observed with the same variables (data not shown). Therefore, the results for the full data set are reported below.

**TABLE 1:** Parameter estimates with standard errors, 95% credible intervals and *P* values (for actual aggression only) for each of actual and net aggression

Model	Variable	Group	Estimate	Std. error	95% CI	*P*
Actual aggression	Genotype	LL	0.36	0.19	0.01 to 0.73	0.048
	Observer	1 v 2	0.79	0.21	0.36 to 1.22	<0.001
		1 v 3	0.47	0.23	0.03 to 0.91	0.035
		2 v 3	–0.31	0.20	–0.70 to 0.07	0.107
	Fish^†^		0.11	0.02		
	Date^†^		0.04	0.06		
	Aquarium^†^		0.03	0.03		
	Family^†^		0.01	0.03		
	Session^†^		0.01	0.01		
	Trial^†^		0.01	0.04		
Net aggression	Genotype	LL	0.12	0.06	0.01 to 0.23	n/a
	Observer	1 v 2	0.01	0.05	–0.10 to 0.11	n/a
		1 v 3	0.01	0.05	–0.10 to 0.10	n/a
		2 v 3	0.00	0.05	–0.10 to 0.10	n/a
	Fish^†^		0.18	0.05		
	Date^†^		0.02	0.02		
	Aquarium^†^		0.02	0.02		
	Family^†^		0.06	0.04		
	Session^†^		0.05	0.06		
	Trial^†^		0.02	0.01		

*Note*: Random effects (†) are shown with their estimates (variance for actual aggression, standard deviation for net aggression) and the standard error of those estimates vgll3*LL genotype is shown relative to vgll3*EE genotype. Observers 2 and 3 are shown relative to observer 1.

Abbreviation: n/a, not available.

### 3.2 Aggression versus colour and feeding activity

**FIGURE 2:**
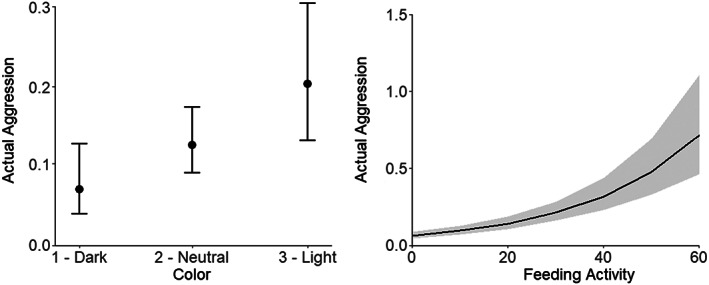
Associations between actual aggression and (a) fish colour and (b) feeding activity. Error bars (left) and the shaded area (right) denote the 95% confidence interval

Individuals with higher actual aggression were significantly lighter in colour and had higher feeding activity during the trials (Table 2 and Figure 2). *Post hoc* testing further showed that light colour was significantly associated with higher aggression than dark colour (estimate = 1.23, SE = 0.38, *Z* = 3.21, *P* = 0.003) or neutral colour (estimate = 0.54, SE = 0.19, *Z* = 2.86, *P* = 0.011), while the difference between dark and neutral colour was in a similar direction, but not significant (estimate = 0.69, SE = 0.36, *Z* = 1.92, *P* = 0.125). There was no significant interaction between feeding activity and colour in predicting aggression.

**TABLE 2:** Parameter estimates with standard errors, 95% credible intervals and *P* values for actual aggression

Variable	Group	Estimate	Std. error	95% CI	*Z*	*P*
Feeding	n/a	0.04	0.01	0.02–0.06	7.68	0.042
Colour	Neutral	0.75	0.37	0.02–1.48	2.04	<0.001
	Light	1.33	0.39	0.57–2.09	3.42	<0.001
Fish^†^		0.28	0.03			
Date		0.05	0.06			
Aquarium		0.04	0.04			
Family		0.01	0.01			
Session		0.01	0.01			

*Note*: Random effects (†) are shown with their overall variance explained (estimate) and its standard error. Neutral and light colours are each shown relative to dark. Feeding activity is continuous.

Abbreviation: n/a, not available.

The same effects tested as predictors of actual aggression were also tested for net aggression, with similar patterns being seen. Specifically, light colour showed significantly higher net aggression than dark colour (estimate = 0.21, SE = 0.08, 95% CI = 0.05–0.37), while neutral colour was associated with higher net aggression than dark colour, but this was not significant (estimate = 0.06, SE = 0.06, 95% CI = −0.06 to 0.19). Feeding activity was also associated with higher aggression (estimate = 0.01, SE <0.01, 95% CI = 0.00–0.01).

### 3.3 *vgll3* genotype versus colour, feeding activity and body condition

Given that lighter fish colour was significantly associated with higher actual aggression, and that vgll3*LL individuals also had higher aggression, we investigated whether there was an association between fish colour and *vgll3* genotype, but this association was not significant (estimate = 0.46, SE = 0.42, 95% CI = −0.41 to 1.26). We similarly investigated whether there was an association between feeding activity and genotype, both of which were also associated with actual aggression in independent analyses, but this association was also not significant (estimate = 0.11, SE = 0.13, 95% CI = −0.17 to 0.37). This indicates that *vgll3* genotype is not explicitly associated with feeding activity or colour, despite all three being associated with aggression. No significant difference between the condition factor between *vgll3* genotypes was observed (estimate = 0.01, SE = 0.02, 95% CI = −0.01 to 0.02).

## 4 DISCUSSION

Aggression has been reported to have a large influence on the growth of fish, with numerous studies showing positive correlations between aggression and growth rate (Buchner *et al*., 2004; Hoogenboom *et al*., 2013; Metcalfe *et al*., 1990; Thorpe *et al*., 1992). It has previously been suggested that earlier maturation in vgll3*EE juveniles is mediated *via* higher body condition, which could possibly be due to increased fat reserves (Debes *et al*., 2021). Given that aggression has a genetic component (Dunbrack *et al*., 1996), we hypothesized that higher aggression in vgll3*EE fish may allow increased food intake and thereby lead to higher body condition. Contrary to our hypothesis, we found that vgll3*LL, and not vgll3*EE, fish displayed increased aggression. Consistent with earlier studies (Keenleyside & Yamamoto, 1962; O'Connor *et al*., 1999; Oikonomidou *et al*., 2019), however, we observed significant associations between fish colour and aggression (lighter coloured fish tended to be more aggressive), and more aggressive individuals also had higher feeding activity, but both of these associations were independent of *vgll3* genotype. Taken together, this suggests that higher body condition previously linked with the vgll3*EE genotype is not directly due to them being more competitive for resources *via* increased aggression and/or feed intake, at least under our experimental scenario.

Given that our initial hypothesis was not supported, how can the higher aggression of vgll3*LL individuals be explained? Although increased aggression may improve access to resources, this may come at a net cost of higher energy expenditure (Réale *et al*., 2010) which, if sufficiently high, could lead to delayed sexual maturation. In natural environments, individuals may calibrate their tendency for aggression with numerous other factors such as the availability of resources, intra‐ and interspecific competition, flow rates and obstacles. These factors may all influence the expected payoff associated with aggression as natural environments are more diverse, and therefore less restrictive, than the experimental laboratory environment, which may allow for more diverse behavioural strategies in the wild (Metcalfe *et al*., 1995; Millinski & Parker, 1991). For example, in the wild, aggressive behaviour may increase the risk of predation (Jakobsson *et al*., 1995); losers of conflicts are able to retreat to avoid injury and excessive aggression may incur metabolic costs in salmon (Puckett & Dill, 1985). This raises the possibility that depending on the context, increased aggression may lead to either net energy loss or net energy gain. Such an explanation would mean that a correlation between aggression and sexual maturation may be context dependent and that our experimental design resulted in aggression, leading to a net expenditure of energy. Thus, future experiments with alternative designs could help to determine the generality of this finding. For example, earlier research reported a negative correlation between aggression and growth rate when food is provided in limited amounts, thus negating the profitability in being aggressive (Vøllestad & Quinn, 2003). Another factor that may have affected the results is dominance hierarchies formed in rearing tanks during the months prior to the experiment. Although individuals from different tanks were used in each trial, and thus were unfamiliar with each other, prior position in a hierarchy could have been a factor. Furthermore, environments with higher flow rates discourage aggression as it increases the cost of attacks (Olla *et al*., 1992). The effects of such factors could be tested by measuring growth and aggression in individuals with different *vgll3* genotypes in low versus high food and/or flow environments, where the profitability of energy expenditure caused by increased aggression may vary depending on the conditions.

As we originally predicted, higher aggression was indeed linked with higher feeding activity. However, this association was independent of *vgll3* genotype. Therefore, the more aggressive vgll3*LL individuals were not always the same individuals with higher feeding activity, suggesting that by diverting energy towards increased aggression, some of the more aggressive vgll3*LL individuals actually spent less time feeding, as has been shown earlier (Fernandes & Volpato, 1993). Furthermore, higher aggression is only expected to have a positive effect on energy budget if the energy intake is higher than the energy used (Laskowski *et al*., 2021). If, for example, more aggressive vgll3*LL individuals also have higher energy use due to them also having higher metabolic rate, or if their dominant status results in them having higher stress levels (Boujard *et al*., 2006; Corrêa *et al*., 2003; Nelissen, 1985), but at the same time they are not succeeding in obtaining more food, then it is possible that by being more aggressive vgll3*LL individuals actually have a reduced energy budget, resulting in a reduced probability to mature early. Future research clarifying the metabolic rate of individuals with differing *vgll3* genotypes would be beneficial in this respect. Finally, our data cannot reveal the proximate mechanism as to why aggressive behaviour at this particular juvenile age/size is associated with *vgll3* genotype. Generally, many behaviours, physiology and life/history traits are assumed to be integrated *via* correlative selection and controlled by complex physiological pathways (Réale *et al*., 2010).

Our findings may also have implications of relevance for aquaculture. Early maturation of fish in aquaculture is generally problematic, as sexual maturation diverts energy away from growing and towards production of gonads and reproductive‐related behaviours, which can lead to reduced growth and flesh quality (Taranger *et al*., 2010). Thus, at first glance, the delayed maturation of phenotype of vgll3*LL fish appears to be desirable for aquaculture. However, this has to be balanced against the new observation reported here of increased aggression displayed by vgll3*LL fish if this result is consistent throughout the salmon life cycle. Thus, although vgll3*LL fish have a higher probability of late maturation, increased aggression, along with the increased social stress that an aggressive vgll3*LL fish may cause on subordinate fish farmed together with it, may counteract some of the benefits that would normally be expected. Aggressive interactions between fish lead to the release of cortisol. Chronic exposure to social stress for subordinate fish leads to a variety of physiological problems such as increased cortisol levels (Pottinger & Carrick, 2001), decreased growth rate (Abbott & Dill, 1989), decreased pathogen resistance (Peters *et al*., 1988) and disease susceptibility (Pottinger & Pickering, 1992). Further studies will be needed to identify the mechanism of how a delayed maturation genotype and aggression are linked, if at all, and whether this finding is consistent throughout the salmon life cycle. Such findings may have implications for aquaculture and further our understanding of the complex nature of the benefits and costs of aggressive behaviour in wild species.

## AUTHOR CONTRIBUTIONS

C.R.P., K.T. and P.B. conceived the study. C.R.P. designed crosses. C.R.P., K.T. and P.B. designed the experimental setup. P.B. and P.L. cared for the fish and designed and constructed the behavioural arenas. P.B., A.T. and K.T. collected the data. C.R.P. coordinated genotyping. P.B. and P.N. performed data analysis. P.B. and C.R.P. drafted the manuscript. All authors provided comments and approved the final version of the manuscript.
